# Outness, Stigma, and Primary Health Care Utilization among Rural LGBT Populations

**DOI:** 10.1371/journal.pone.0146139

**Published:** 2016-01-05

**Authors:** J. Whitehead, John Shaver, Rob Stephenson

**Affiliations:** 1 Department of Pediatrics, School of Medicine, Northwestern University, Chicago, IL, United States of America; 2 Department of Health and Biological Sciences, School of Nursing, University of Michigan, Ann Arbor, Michigan, United States of America; University of Toronto, CANADA

## Abstract

**Background:**

Prior studies have noted significant health disadvantages experienced by LGBT (lesbian, gay, bisexual, and transgender) populations in the US. While several studies have identified that fears or experiences of stigma and disclosure of sexual orientation and/or gender identity to health care providers are significant barriers to health care utilization for LGBT people, these studies have concentrated almost exclusively on urban samples. Little is known about the impact of stigma specifically for rural LGBT populations, who may have less access to quality, LGBT-sensitive care than LGBT people in urban centers.

**Methodology:**

LBGT individuals residing in rural areas of the United States were recruited online to participate in a survey examining the relationship between stigma, disclosure and “outness,” and utilization of primary care services. Data were collected and analyzed regarding LGBT individuals’ demographics, health care access, health risk factors, health status, outness to social contacts and primary care provider, and anticipated, internalized, and enacted stigmas.

**Results:**

Higher scores on stigma scales were associated with lower utilization of health services for the transgender & non-binary group, while higher levels of disclosure of sexual orientation were associated with greater utilization of health services for cisgender men.

**Conclusions:**

The results demonstrate the role of stigma in shaping access to primary health care among rural LGBT people and point to the need for interventions focused towards decreasing stigma in health care settings or increasing patients’ disclosure of orientation or gender identity to providers. Such interventions have the potential to increase utilization of primary and preventive health care services by LGBT people in rural areas.

## Introduction

Healthy People 2020 [1] identified increasing access to quality healthcare for LGBT (lesbian, gay, bisexual, and transgender) populations as a priority for further research and intervention. Health disparities affecting LGBT people are well-documented, and span each subgroup of this population. Among these disparities is increased prevalence of a number of risk factors for poor health. For example, lesbian women have repeatedly been shown to have higher alcohol intake [2–3] and higher BMIs [3] than heterosexual women, while gay and bisexual men have been reported as having higher average markers of cardiovascular stress [3] and higher incidence of illicit drug use [4] than heterosexual men. LGBT populations overall have higher rates of tobacco use than their heterosexual, cisgender (those who identify their current gender as that assigned to them at birth) counterparts [5]. Additionally, some LGBT individuals incur increased risk through behaviors that can be associated with LGBT identity. These behaviors include hormone therapy among certain transgender persons, which can increase risk for cardiovascular conditions and organ damage [6], and receptive anal intercourse, which has been associated with increased risk for certain anal cancers [7], HIV [8], and other sexually transmitted infections [9] among gay and bisexual men.

This augmented prevalence of risk factors is confounded by LGBT people’s decreased access to care and lower rates of screenings for multiple preventable or treatable diseases [10]. LGBT adults are significantly less likely than non-LGBT adults to have a PCP [11]. Despite advances seen through the Affordable Care Act, LGBT people are also more likely to report being uninsured [12] and unable to afford health services [13] than their non-LGBT peers.

Lower rates of primary care utilization among LGBT populations may be founded on expectations and experiences of stigma based on sexuality or gender identity. This stigma can be defined as “the negative regard, inferior status, and the relative powerlessness that society collectively accords to any non-heterosexual [or non-cisnormative] behavior, identity, relationship, or community” [14]. Sexuality- and gender-identity-based stigma has been widely cited as influential over substance abuse [15], sexual risk behavior such as unprotected anal intercourse [8, 16], health care utilization [17], and decreased preventive care including cancer screenings [18] among LGBT populations. As represented by the Minority Stress Model, the stigma and stress experienced by LGBT individuals contribute to the determination of health for LGBT individuals [19–20]. According to the Model, general environmental elements (such as socioeconomic status or geographic location) and minority status (such as LGBT community label) determine exposure to stressors affecting health [19–20]. These stressors can be general (such as divorce or home foreclosure) or minority-status-related (such as homophobic discrimination at work) [19–20]. Identification with a community label (such as the term “gay” and the “gay community”) can lead to additional personal stressors (such as devalued self-perception) and can influence one’s coping mechanisms related to minority stressors (such as avoiding accessing health care that would require disclosure of lesbian identity) [19–20]. Health outcomes are often more dramatically impacted with more intense identification with a LGBT community label [19]. Contrarily, identification with a community label can add community-specific resources to one’s coping abilities (such as a transgender man’s social support from other transgender men) that can decrease the impacts of minority stress [19–20]. Each of these elements culminate in the health outcomes of the LGBT individual [19–20].

Stigma can be understood as having three domains: anticipated stigma (concern for possible future instance of discrimination), internalized stigma (devaluation of self, based on sexual orientation or gender identity), and enacted stigma (actual instances of experienced discrimination) [21]. Each type of stigma may impact health-related behavior in a unique manner. For example, anticipated stigma may cause a patient to avoid or delay accessing clinical care settings, as they are potentially discriminatory [22]. Internalized stigma has been correlated with lowered self-esteem, which increases the potential for participation in negative health behaviors [21, 23–24]. Instances of enacted stigma can lead to poorer mental health, making it difficult for a patient to access care [25]. Each of these types of stigma have been documented across LGBT populations; however, transgender patients report notably higher rates of maltreatment in healthcare encounters, including denial of care [26], with resultant uncertainty that future providers will know how to treat them appropriately.

Stigma also interacts with and effects “outness,” or disclosure of one’s sexual orientation and/or gender identity to social contacts. Increasing levels of stigma are correlated with decreased levels of disclosure of sexual orientation and/or gender identity to health care providers [27]. Prior studies have found widely varying rates of disclosure to health care providers, from 50% of non-urban lesbians [27–28], to 90% of gay men in an urban center [29], to 43% of transgender patients in a single state [17]. The connection between disclosure and increased preventive care for gay and bisexual men is fairly direct, as specific recommendations exist for HIV screening as well as hepatitis A and B vaccinations based on sexual behavior [30–31]. However, increased utilization of this type of preventive care assumes that providers are aware of the behavior-specific recommendations, which is not always the case [31]. Interestingly, prior studies have shown that women who disclose their sexual orientation are more likely to have obtained Pap smears [32–33], despite the fact that Pap smear recommendations do not differ based on sexual orientation. In this case, lack of disclosure of sexual orientation may be associated with a general discomfort discussing other sensitive topics with health care providers [34], such as substance use or medication adherence [35]. Thus, unwillingness to discuss sexual orientation due to fear of discrimination or judgment may lead to missed opportunities for other types of preventive care counseling or screening discussions.

Historically, the majority of studies of LGBT populations have been conducted in urban centers due to ease of access to high concentrations of LGBT people in social spaces catering to this community. Though little data exists thus far on the rural LGBT population, at first glance rural areas appear less hospitable, in part due to increased stigma and social isolation [36]. Even non-LGBT rural populations face a complex set of barriers to health care that includes geography, lack of transportation, lack of finances or insurance, and provider shortages [37–40]. While disclosure of orientation or identity in rural communities may increase access to social support and appropriate healthcare recommendations, this may also increase the risk of discrimination and stigma [41].

In order to facilitate increased access to rural LGBT populations, the internet has emerged as an effective method of study recruitment. Although racial and socio-economic status-based biases remain concerns in internet surveying, these concerns are diminishing as internet access becomes more widespread [42]. A recent study comparing a sample of men who have sex with men recruited online to a sample recruited using venue time-based sampling found few differences in the two populations as well as reduced cost associated with online recruitment [43]. The current study used an internet-recruitment method to survey a nationwide sample of rural LGBT people. Though previous studies have shown separately that rural LGBT individuals may be less likely to access primary care, may experience more stigma, or may come out less often to their providers, prior studies have not investigated the interactions between each of these factors that contribute to health care disparities. The aim of this study is to determine whether higher levels of stigma and/or lower levels of outness correlate with less primary health care access for rural LGBT populations. Because our sample contains a wide spectrum of sexual orientations and gender identities, we will also attempt to highlight differences in stigma, outness, and access to care between these groups.

## Methods

Participants were recruited to the online survey primarily via banner ads on Facebook during a 17 day period in August 2014. Ads were targeted towards Facebook users age 18+ with LGBT-related interests who reported residence in rural zip codes (based on the U.S. Census Bureau’s definition of areas with a population density of <1,000 people per square mile [44]. Clicking on the advertisement took participants to the informed consent page of the survey, which contained information about the purpose of the study, the voluntary and anonymous nature of the study, and the HIPAA privacy rules. Participants provided electronic informed consent. No incentive was offered for participating in the survey, which took an average of 20 minutes to complete. The study was approved by the Emory University IRB.

Responses to the initial five questions (age over 18; rural home zip code; and self-identification as LGBT based on sex assigned at birth, current gender identity, and sexual orientation) determined eligibility for the study.

Healthcare utilization was assessed by asking the frequency of visits (number of visits to various health care providers in the past 12 months) [45], health insurance status, and whether the participant had a PCP (*“Do you have one location where you typically go to receive primary care services*?” and *“Do you have one person you think of as your primary doctor or health care provider for checkups*, *medication refills*, *or treatment for non-emergent illnesses*?*”*). Participants were asked follow-up questions about how they chose their PCP and how LGBT-friendly they perceived their provider to be. For participants who indicated that they did not have a PCP, they were asked to respond to questions with regards to the provider they had seen the most often in the last year.

General health status was assessed in two ways: by self-report (*excellent*, *very good*, *good*, *fair*, or *poor*) [46] and by whether participants self-reported that they had been diagnosed with any chronic disease. Symptoms of possible clinical depression were measured using the CES-D 11-item Iowa form [47–48].

Our main measure of interest in determining primary care utilization was whether participants were up to date on age- and anatomy- appropriate vaccinations and health screenings that are generally obtained in, or referred to from, a primary care setting. We used the U.S. Preventive Services Task Force & Centers for Disease Control guidelines to compile a list of the minimum recommended health care tasks for the general asymptomatic population and targeted each question to the participant’s age, sex assigned at birth, and, in some cases, sexual orientation or other risk factors. For questions where the sex assigned at birth may not match a participant’s current anatomy, participants were given the option to note that the question does not apply to them (ex: prior surgery to remove breasts or cervix, thus obviating the need for screening). Recommended vaccinations included influenza [49], tetanus [49], HPV [49], Shingles [49], hepatitis A [49], and hepatitis B [49]. Health screenings included tests to detect high blood pressure [50], HIV [30], gonorrhea [51], chlamydia [52], cervical cancer [53], high cholesterol [54], hepatitis C [55], breast cancer [56], colon cancer [57], osteoporosis [58], and abdominal aortic aneurysms [59].

To model factors associated with primary care utilization, we determined the criteria for being “up to date” on each health screening or vaccination, and a categorical variable for whether respondents met these criteria was created for each health task. Respondents who indicated they did not know if or when they had completed the health task were deemed not up to date. A singular outcome variable, the “Health Score,” was created to represent the percentage of health tasks each respondent had obtained within the recommended time period. For example, recommendations for an 18-year-old participant include an HIV test and gonorrhea/chlamydia testing (if sexually active), blood pressure measurement, and vaccinations against the flu, HPV, and tetanus. If the participant received at least one dose of the HPV vaccine [60], had a tetanus vaccine within the last ten years, and had her blood pressure measured within the last year (3 of the 6 recommended tests), her Health Score would be 0.50.

In order to measure stigma and disclosure, participants were asked to select or type in an identifying label for themselves (such as *gay* or *queer*), and the survey sections on stigma and disclosure were coded to fill in their response throughout (ex: “I have wished I was not *queer”*). Stigma was measured in three domain, with responses limited to experiences of stigma in the last 12 months that were specifically related to sexual orientation and/or gender identity. The stigma scales used were abbreviated from Meyer to measure only stigma [61], and each scale utilized has shown good reliability and validity in previous testing with lesbian, gay, and bisexual populations of various racial and ethnic identities [62]. Internalized stigma was measured by seven items rated *Often*, *Sometimes*, *Rarely*, or *Never* (ex: “*I have felt that being queer is a personal shortcoming”*). Enacted stigma (actual experiences of everyday stigma or discrimination) was measured using 14 items, four of which were specific to experiences in healthcare settings [63]. Respondents indicated whether each event had happened *Never*, *Once*, *2–3 times*, or *4+ times* (ex: “*I have been rejected by family members”)*. Anticipated stigma was measured using six items, rated *Strongly Agree* to *Strongly Disagree* (ex: “*Most people look down on queer people”*). Outness was measured using a standardized scale of outness to eleven social contacts or groups [64]. An additional question measured outness to the participant’s PCP (or provider seen most often) on the same rating scale for comparison.

Respondents were divided into three groups for analysis: cisgender men (those assigned male at birth who identify their current gender as male and sexual orientation as something other than straight/heterosexual), cisgender women (those assigned female at birth who identify their current gender as female and sexual orientation as something other than straight/heterosexual), and transgender & non-binary (those who currently identify as transgender, or as any gender that does not exactly match their sex assigned at birth, of any sexual orientation).

Key exposures for analysis were Outness to PCP (range 1–7) and “Average Outness” to social contacts (range 1–7), a variable created by dividing the total score on the Outness Inventory by the number of items answered, since items may not apply to all individuals (ex: “*my religious community”*). Other key exposures were the three domains of stigma: additive scores on each domain scale were divided into three categories representing scores below approximately the 25^th^ percentile, approximately 25^th^-75^th^ percentile, and approximately 75^th^ percentile and above. This division determined whether a dose-response relationship was present between reported stigma values and decreased health outcomes. Percentiles were based on the distribution of scores in the overall respondent group and are not exact due to the uneven score distribution. Other covariates included age, race, insurance status, depression, relationship status, employment status, education level, presence of chronic disease, smoking history, alcohol use, general health self-report, and travel time to PCP. Descriptive data analysis and fitting of data to a generalized linear regression model were completed using STATA 13.1. In addition, chi-squared and Kruskal-Wallis tests were used to determine if differences between groups were significant.

A total of 1,018 competed survey responses (477 cisgender men, 368 cisgender women, and 169 from transgender & non-binary individuals) were obtained and are incorporated into the descriptive data tables. Of these, 946 responses (451 cisgender men, 340 cisgender women, and 155 transgender & non-binary individuals) were included in data analysis, as 4 responses from intersex individuals were dropped due to small sample size and 68 responses were dropped due to missing data in the key variables utilized in analysis. The total data set was obtained through 17 days of banner advertisement on Facebook, yielding 972 completed responses, with an additional 24 surveys from a Tumblr site posting, 18 referrals from survey participants, and 4 responses from a listserv of transgender individuals primarily in the Southeast. Eight hundred and ninety-one other participants who attempted the survey were disqualified for not meeting eligibility criteria of rural residency, age ≥18, and self-identification as non-cisgender or non-heterosexual.

## Results

The mean age of the respondents was 32, though 41% of the sample fell in the 18–24 age category (Table 1). The sample was 88% white and 91% non-Hispanic. This compares with the overall population of rural America that is 78% white and 91% non-Hispanic [65]. Eighty-six percent and 95% of the cisgender women and men, respectively, identified as “lesbian, gay or homosexual.” Cisgender women were more likely to cohabit (41%) than the other two groups, while cisgender men were more likely to be single (59%). The respondents overwhelmingly reported access to the internet in their home or on their mobile device, with only 3–4% reporting primary use at a public location or a friend/family member’s house.

**Table 1:** Demographic Characteristics of an online sample of rural LGBT people.

	Cisgender Women (n = 368)	Cisgender Men (n = 477)	Transgender & Non-binary Persons (n = 169)	All (n = 1014)
**Age**				
Mean +/- StdDev (range)	32.26+/- 12.76 (18–76)	32.62 +/-13.42 (18–75)	32.20 +/- 14.09 (18–73)	32.42+/-13.29 (18–76)
**Race**^**1**^				
Black	16 (4%)	6 (1%)	4 (2%)	26 (3%)
White	320 (87%)	425 (89%)	151 (89%)	896 (88%)
Other	30 (8%)	45 (9%)	12 (7%)	87 (9%)
**Ethnicity**				
Non-Hispanic	337 (92%)	435 (91%)	155 (92%)	927 (91%)
Hispanic	25 (7%)	33 (7%)	10 (6%)	68 (7%)
**Orientation**^**2**^				
Gay/Lesbian/Homosexual	318 (86%)	452 (95%)	56 (33%)	826 (81%)
Bisexual	27 (7%)	11 (2%)	25 (15%)	63 (6%)
Straight/Heterosexual	0 (0%)	0 (0%)	26 (15%)	26 (3%)
Queer	12 (3%)	9 (2%)	34 (20%)	55 (5%)
Something Else	11 (3%)	5 (1%)	28 (17%)	44 (4%)
**Relationship Status**				
Single	122 (33%)	283 (59%)	77 (46%)	482 (48%)
Cohabiting	150 (41%)	118 (25%)	36 (21%)	304 (30%)
Married/Divorced/Widowed	94 (26%)	75 (16%)	56 (33%)	225 (22%)
**Education**				
High School or lower	76 (21%)	121 (25%)	29 (17%)	226 (22%)
Some college or Associate's/Technical Degree	189 (51%)	239 (50%)	91 (54%)	519 (51%)
Bachelor's Degree or higher	100 (27%)	117 (25%)	48 (28%)	265 (26%)
**Internet Access**				
At home	188 (51%)	299 (63%)	117 (69%)	604 (60%)
Family/Friend's/Public	11 (3%)	21 (4%)	5 (3%)	37 (4%)
Cell network	167 (45%)	157 (33%)	46 (27%)	370 (37%)

^1^Other races include Asian, Pacific Islander, Native American/Alaskan Native/Inuit, Multiracial.

^2^When specifying sexual orientation, participants could select "Something Else" and enter free text.

N (%) unless otherwise specified.

Insurance status was similar across all groups (82% overall) (Table 2). Eighty-three percent reported using one location (i.e., one medical practice) as their main source of primary care, and 74% reported they had one provider within this practice that they considered to be their PCP. Percentage of respondents reporting a routine check-up within the last two years varied significantly across groups, and was highest among cisgender women (82%). Similarly, cisgender women were more likely to have visited their PCP for any reason, while 30% of cisgender men had not seen their PCP in the last year.

**Table 2:** Reporting of Health Care Access and Utilization of an online sample of rural LGBT people.

	Cisgender Women (n = 368)	Cisgender Men (n = 477)	Transgender & Non-binary Persons (n = 169)	All (n = 1014)	P^5^
**Health Insurance**					
	305 (83%)	393 (82%)	137 (81%)	835 (82%)	0.845
**Single PCP Location**^**1**^					
	312 (85%)	396 (83%)	138 (82%)	846 (83%)	0.628
**Single PCP**^**2**^					
	283 (77%)	349 (73%)	116 (69%)	748 (74%)	0.119
**Routine Check-up**^**3**^					
	302 (82%)	360 (75%)	125 (74%)	787 (78%)	**0.034**
**Travel time >1hr to main provider**^**4**^					
	20 (5%)	25 (5%)	24 (14%)	69 (7%)	**0.000**
**Average “outness” to personal contacts**^**5**^					
Mean +/- StdDev	5.12 +/- 1.48	5.10 +/- 1.37	4.28 +/- 1.34	4.97 +/- 1.44	**0.000**
**Average “outness” to main provider**^**5**^					
Mean +/- StdDev	4.47 +/- 2.27	4.52 +/- 2.33	4.88 +/- 2.51	4.56 +/- 2.34	0.1062
**Ranked “Provider sees other LGBT patients” in top 3 reasons for choosing provider**					
	29 (8%)	30 (6%)	46 (27%)	105 (10%)	N/A
**Importance of provider having LGBT-specific knowledge & support** Scale of 1 (not important) to 5 (very important)					
Mean +/- StdDev	3.90 +/- 1.15	4.11 +/- 1.06	4.57 +/- 0.81	4.11 +/- 1.08	0.000
**Primary Care Provider Visits**^**6**^					
0	73 (20%)	142 (30%)	36 (21%)	251 (25%)	**0.005**
1–2	136 (37%)	174 (36%)	64 (38%)	374 (37%)	** **
3+	158 (43%)	157 (33%)	67 (40%)	382 (38%)	** **
**One or more visits to an LGBT-specific clinic**^**6**^					
	7 (2%)	18 (4%)	19 (11%)	44 (4%)	**0.000**

^1^Has one office location where primary care services are obtained.

^2^Has one single provider that they consider to be their primary care provider (PCP).

^3^Has had a routine check-up/physical exam within the last 2 years.

^4^For most, their main provider is their PCP, but for those without a PCP, it indicates the provider they visited most often within the last year.

^5^The chi-squared test or the Kruskal-Wallis test was used to determine significant differences between each of the 3 gender categories.

^6^Self-reported visits in the last 12 months.

On a scale of 1–7 [64], respondents reported an average outness to personal contacts of 4.97, with the transgender & non-binary group having significantly less outness on average (p = 0.0001). Although outness to main health care provider did not vary significantly across groups, transgender & non-binary respondents were over three times more likely to choose “This provider sees other LGBT patients” as one of their top three reasons for choosing that provider, and also rated the importance of their provider having LGBT-specific knowledge significantly higher than their cisgender counterparts (p = 0.0001). Transgender & non-binary respondents were also almost three times as likely as the cisgender groups to report traveling over one hour to their PCP’s office (14% vs. 5%), possibly related to the fact that in the last year, one in ten had visited an LGBT-specific health care clinic, which are often located in urban areas.

On average, cisgender men reported their general health status to be better than both of the other groups (p = 0.0001) (Table 3). However, cisgender men actually had significantly lower objective scores of the percent of health screenings obtained (55% for cisgender men, 62% for cisgender women, 58% for transgender & non-binary individuals; p = 0.001). Overall, 50% of the population met criteria on the CES-D for symptoms that may indicate clinical depression, and transgender & non-binary respondents had a significantly higher rate (65%) of self-reported depression symptoms than the other two groups (p = 0.0001). Approximately one-third of respondents in each group had been diagnosed with one or more chronic diseases. In terms of health risk factors, 40% of the overall sample currently smokes tobacco, and an additional 21% have at least some smoking history but have since quit. Fourteen percent of the population met criteria for high-risk drinking (defined as ≥8 drinks/week for those assigned female at birth or ≥15 drinks/week for those assigned male at birth [66]), though transgender & non-binary respondents had significantly lower rates of risky alcohol use (p = 0.038) and present tobacco use (p = 0.0000) than either cisgender group. Data on binge drinking episodes was not collected. Regarding the stigma scales, the mean score on each subscale was as follows: anticipated, 10.62 +/- 6.36 (range 0–24); enacted, 8.10 +/- 8.24 (range 0–42); internalized, 3.72 +/- 4.12 (range 0–21). Though no standardized means exist, it is interesting to note that while cisgender men and women had similar scores across all scales, transgender & non-binary respondents consistently reported higher levels of all forms of stigma (p = 0.000).

**Table 3:** Health Status and Risk Factors of an online sample of rural LGBT people.

	Cisgender Women (n = 368)	Cisgender Men (n = 477)	Transgender & Non-binary Persons (n = 169)	All (n = 1014)	P^2^
**Health Status Self-Report**					
Mean +/- SD	3.18 +/- 1.04	3.45 +/- 0.94	3.15 +/- 0.99	3.3 +/- 0.99	**0.000**
**Average Health Score**					
Mean +/- StdDev (Range 0–1)	.619 +/- .247	.551 +/- .262	.577 +/- .253	.579 +/- .257	**0.001**
**CES-D Depression Score**					
Mean +/- StdDev (Range 0–22)	9.00 +/- 4.99	7.69 +/- 4.92	10.34 +/- 5.54	8.60 +/- 5.14	**0.000**
% of scores >9 (indicating possible clinical depression)	198 (54%)	195 (41%)	109 (65%)	502 (50%)	**0.000**
**Chronic Disease**					
Diagnosed with one or more chronic diseases	140 (38%)	154 (32%)	59 (35%)	353 (35%)	0.197
**Tobacco Use**					
Current Smoker	164 (45%)	191 (40%)	53 (31%)	408 (40%)	**0.000**
Former Smoker	82 (22%)	83 (17%)	52 (31%)	217 (21%)	
Never Smoked	121 (33%)	201 (42%)	64 (38%)	386 (38%)	
**Alcohol Use**					
Heavy Alcohol Consumer^1^	47 (13%)	76 (16%)	14 (8%)	137 (14%)	**0.038**
**Anticipated Stigma**					
Mean +/- StdDev (Scale 0–24)	9.75 +/- 6.09	9.98 +/- 6.23	14.31 +/- 6.04	10.62 +/- 6.36	**0.000**
**Enacted Stigma**					
Mean +/- StdDev (Scale 0–42)	7.50 +/- 8.25	7.06 +/- 7.46	12.28 +/- 9.03	8.10 +/- 8.24	**0.000**
**Internalized Stigma**					
Mean +/- StdDev (Scale 0–21)	2.93 +/- 3.57	3.29 +/- 3.54	6.64 +/- 5.33	3.72 +/- 4.12	**0.000**

^1^Heavy alcohol use was defined as 8+ drinks/week for those assigned female at birth, or 15+ drinks/week for those assigned male at birth. Assigning groups for number of drinks/week based on current hormone profile produced similar percentages but with greater uncertainty due to missing hormone data.

^2^The chi-squared test or the Kruskal-Wallis test was used to determine if differences between groups were significant.

Data on uptake of recommended vaccines and health screenings from this sample show that the percentage of respondents up to date on individual health tasks ranged from a low of 26% for the HPV vaccine to a high of 100% for osteoporosis screening. Table 4 provides details on each recommendation.

**Table 4:** Uptake of Recommended Health Screenings & Vaccinations of an online sample of rural LGBT people.

Vaccination or Screening	Question Criteria^1^	Criteria to be Up-To-Date	N^2^	% Up-To-Date
Influenza vaccine	All genders; all ages	Received vaccine (either nasal, intradermal, or intramuscular) within the last 12 months	1011	41
Tetanus vaccine	All genders; all ages	Received Tdap, Td, or Dtap within the last 10 years	1011	69
HPV vaccine	All genders; age 18–34	Received at least 1 dose at any time point	660	26
Shingles vaccine	All genders; age 60+	Received vaccine at any time point	45	36
Hepatitis A vaccine	Assigned male at birth; reporting a combination of current gender identity and sexual orientation that indicate that the respondent is interested in men.^3^	Received vaccine at any time point	516	41
Hepatitis B vaccine	Assigned male at birth; reporting a combination of current gender identity and sexual orientation that indicate that the respondent is interested in men.^3^	Received vaccine at any time point	516	49
High Blood Pressure Screening	All genders; all ages	Blood pressure measured within the last 12 months	1012	92
HIV Screening	All genders; all ages	Screened (via cheek swab, finger prick, or blood test) within the last 12 months, or selected a legitimate reason for not being screened (ie, no sexual activity within the last 12 months or already HIV positive)	1008	57
Gonorrhea/ Chlamydia Screening	All genders; age 18–24	Screened (via urine, genital or rectal sample) at any time point, or selected "I have never had sex"	351	45
Cervical Cancer Screening	Assigned female at birth; age 21–65	Pap smear within the last 3 years if age 21–30, within the last 5 years if age 30–65, or selected "I do not have a cervix"	357	69
High Cholesterol Screening	Assigned male at birth; age 35+	Blood test at any time point	207	92
Hepatitis C Screening	All genders; age 48–69	Blood test at any time point	159	56
Breast Cancer Screening	Assigned female at birth, OR assigned male at birth and has ever taken hormones to reflect gender identity; age 50–74	Mammogram within the last 2 years, or selected "I do not have breast tissue"	60	62
Colon Cancer Screening	All genders; age 50–75	Colonoscopy within the last 10 years, sigmoidoscopy within the last 5 years, or fecal occult blood test within the last year	137	66
Osteoporosis Screening	Assigned female at birth; age 65+	DEXA scan at any time point	5	100
Abdominal Aortic Aneurysm Screening	Assigned male at birth; age 65–75; current or former smoker	Abdominal ultrasound at any time point	13	38

^1^Criteria used by the survey code to determine whether each participant should be shown each question. (Ex: The question about the Shingles vaccine was hidden for all respondents under the age of 60).

^2^The number of respondents who a) fit the criteria to be shown the question, and b) provided a response.

^3^Data on sexual behavior was not collected.

Table 5 shows the results of the regression model. Being insured was significantly associated with higher health scores for the population overall (β = 0.362, SE = 0.095, p = 0.000) and for both cisgender groups (women: β = 0.362, SE = 0.142, p = 0.011; men: β = 0.320, SE = 0.140, p = 0.022), but not for the transgender & non-binary group (β = 0.226, SE = 0.223, p = 0.311). Depression (scoring >9 on the CES-D) was significantly associated with worse health scores for cisgender women only (β = -0.275, SE = 0.111, p = 0.013). Higher levels of education were associated with higher health scores for the sample overall (some college: β = 0.194, SE = 0.083, p = 0.020; college degree or higher: β = 0.282, SE = 0.100, p = 0.005). Being diagnosed with a chronic disease was associated with higher health scores for all groups except the transgender & non-binary group (overall sample: β = 0.336, SE = 0.077, p = 0.000; cisgender women: β = 0.241, SE = 0.121, p = 0.046; cisgender men: β = 0.547, SE = 0.119, p = 0.000), likely due to increased retention in care. For cisgender women, being a former smoker was associated with higher health scores (β = 0.328, SE = 0.164, p = 0.045). For all groups except the transgender & non-binary participants, being out to providers was associated with higher health scores (overall sample: β = 0.093, SE = 0.017, p = 0.000; cisgender women: β = 0.112, SE = 0.029, p = 0.000; cisgender men: β = 0.119, SE = 0.026, p = 0.000). For cisgender women, being in the middle 50% on scores of internalized homophobia was actually significantly associated with higher health scores (compared to either the bottom quartile or the top quartile; β = 0.297, SE = 0.126, p = 0.018). For cisgender men and women, neither enacted nor anticipated stigma scores consistently predicted overall health scores. However, for transgender & non-binary respondents, higher scores on enacted stigma (middle 50%: β = -0.663, SE = 0.286, p = 0.020; top 25%: β = -0.772, SE = 0.338, p = 0.022) or anticipated stigma (top 25%: β = -0.731, SE = 0.372, p = 0.049) predicted significantly worse overall health scores.

**Table 5:** Results of Linear Regression Model of factors associated with Health Care Utilization of an online sample of rural LGBT people.

	Cisgender Females			Cisgender Males			Transgender & Non-binary Persons			All		
	Coeff	Std Error	P value	Coeff	Std Error	P value	Coeff	Std Error	P value	Coeff	Std Error	P value
**Insurance Status**												
Uninsured	Ref			Ref			Ref			Ref		
Insured	0.362	0.142	0.011	0.320	0.140	0.022	0.226	0.223	0.311	0.362	0.095	0.000
**CES-D Depression Score**												
<9	Ref			Ref			Ref			Ref		
≥9	-0.275	0.111	0.013	-0.003	0.107	0.978	0.260	0.191	0.174	-0.075	0.072	0.299
**Education**												
High school diploma or less	Ref			Ref			Ref			Ref		
Some college	0.080	0.149	0.595	0.246	0.115	0.032	0.586	0.226	0.009	0.194	0.083	0.020
College degree or higher	0.197	0.175	0.261	0.388	0.144	0.007	0.636	0.277	0.022	0.282	0.100	0.005
**Chronic Disease**												
No chronic disease	Ref			Ref			Ref			Ref		
1+ Chronic disease	0.241	0.121	0.046	0.547	0.119	0.000	0.229	0.210	0.274	0.336	0.077	0.000
**Smoking History**												
Never smoked	Ref			Ref			Ref			Ref		
Past smoker	0.328	0.164	0.045	-0.150	0.136	0.270	0.126	0.237	0.595	0.074	0.094	0.429
Current smoker	0.117	0.130	0.369	-0.121	0.108	0.263	0.018	0.213	0.933	-0.021	0.077	0.790
**Outness to PCP**												
	0.112	0.029	0.000	0.119	0.026	0.000	0.000	0.042	0.998	0.093	0.017	0.000
**Internalized Stigma**												
Bottom 25%	Ref			Ref			Ref			Ref		
Middle 50%	0.297	0.126	0.018	-0.023	0.114	0.839	0.407	0.263	0.121	0.154	0.080	0.053
Top 25%	0.191	0.155	0.217	-0.171	0.149	0.252	0.301	0.269	0.262	0.035	0.100	0.724
**Enacted Stigma**												
Bottom 25%	Ref			Ref			Ref			Ref		
Middle 50%	-0.023	0.124	0.851	0.087	0.116	0.456	-0.663	0.286	0.020	-0.012	0.080	0.885
Top 25%	0.052	0.170	0.760	0.395	0.166	0.017	-0.772	0.338	0.022	0.137	0.108	0.204
**Anticipated Stigma**												
Bottom 25%	Ref			Ref			Ref			Ref		
Middle 50%	0.124	0.138	0.370	0.027	0.111	0.805	-0.633	0.344	0.066	0.029	0.086	0.733
Top 25%	0.133	0.180	0.458	0.104	0.151	0.489	-0.731	0.372	0.049	-0.011	0.108	0.919

## Discussion

These results illustrate a high prevalence of risk factors for poor health among this online sample of rural LGBT, including high rates of smoking and binge drinking. Self-reported symptoms consistent with depression were highly prevalent in our sample (50%); this compares with rates of 13% for adults and 25% for youth in a recent survey of depressive symptoms among sexual and gender minorities recruited at urban Pride festivals [67]. Depression has previously been associated with primary care underutilization [68]. Similarly, depression scores for cisgender women in this study were significantly associated with lower primary health care utilization. For cisgender women, poor mental health may be shaping a lack of access to primary health care or may be a product of a lack of access to health care.

This sample’s high prevalence of risk factors for poor health and high incidence of pre-existing chronic disease (35%) are concerning in combination with reported low uptake in our sample of recommended vaccinations and screening tests for preventable diseases. Clearly, a notable portion of our rural LGBT sample has not met recommended primary care utilization for healthy people. The relationships between risk factors, chronic disease, and poor prevention behaviors in LGBT individuals warrants further investigation.

Members of each subgroup (cisgender men, cisgender women, and transgender & non-binary individuals) reported experiencing each form of stigma. The transgender & non-binary participants in this study, however, reported much higher levels of all three types of stigma. Not only does this group experience greater stigma, but our data reveal that higher anticipated and enacted stigma scores in this group were significantly associated with lower self-reported health scores, while there was no significant correlation between stigma and health score for both cisgender groups. It may be that stigma directly decreases care-seeking behavior among transgender & non-binary participants, for instance, due to fear of future discrimination or anger regarding past discrimination; further research is needed to clarify this association. With the recent increase in legal protections, health care knowledge, and public visibility of transgender people, stigma associated with being transgender may gradually reduce, allowing, in turn, for increases in health. In the meantime, strategies that have been shown to overcome stigma and improve health care access in rural areas must be employed. Methods that have proven effective include specialist consultation via telemedicine [69], specialist mobile outreach clinics [70], and increased primary care provider training on the specificities of health care for transgender individuals [6, 71–74]. Implementing these strategies can reduce health care stigma and uncertainty, improving access to quality health care for rural transgender Americans.

Degree of outness to health care providers was significantly associated with increased primary care utilization for cisgender men and women. For men, this correlation can be somewhat direct as there are specific vaccination recommendations based on sexual behaviors (often but not always related to sexual orientation). It may also be that outness is correlated with better rapport between patient and provider, or greater attentiveness of the provider to holistic care (including preventive care). Training providers on LGBT health concerns and on how to make their practices communicate LGBT-friendliness may allow for increased patient comfort in disclosure and greater patient-provider rapport. Practices that have been associated with positive patient-provider relations and optimized opportunity for LGBT people to disclose include intake forms and interviews that do not assume cisgender or heterosexuality [71, 73, 75–76], basic provider knowledge of legal and cultural issues facing LGBT people [71–72, 74–75, 77–78], awareness by all staff of LGBT-specific health concerns [6, 72, 74, 77–78], and displayed non-discrimination policy including protections based on sexual orientation and gender identity [72, 74, 77–78].

For transgender & non-binary respondents, no correlation between outness and greater utilization of care was found, which may indicate that the outness that would generally be associated with more individualized health recommendations may instead be negated by another factor, such as a lack of knowledge on the provider’s part that reduces the likelihood of preventive health recommendations. However, outness did not appear to be entirely dependent on the provider asking the appropriate questions to elicit disclosure. In our overall sample, of the 37% who were not out to their main provider, only 28% reported feeling that their provider might be uncomfortable with their disclosure. Forty-three percent felt their sexual orientation or gender identity had “no bearing on [their] health” and 27% said it was “none of [the provider’s] business.” Thus, vocalizing and providing openly available materials regarding the benefits of disclosure and the relationship between sexual behavior and overall health [72, 74, 76–78], may be the most potent in increasing health for some LGBT patients.

Degree of rurality is another factor that requires further study and explanation. In our sample, transgender & non-binary respondents were more likely to travel longer distances to receive care, possibly related to their propensity to prioritize and seek out more LGBT-specific care (again, possibly related to this group’s higher levels of experienced stigma). Many of the vaccination and screening recommendations require separate or repeated visits to a health care provider (for example, the 3-dose HPV vaccine series, or a colonoscopy which requires an escort to transport the patient after the procedure). Long travel times may reduce the rural LGBT individual’s ability to utilize primary care clinics, and this effect seemed to be exaggerated in our transgender & non-binary population.

Insurance status surely impacts access to care in the general population, and that held true for our sample of rural cisgender men and women. However, insurance status did not significantly correlate with greater utilization of primary care for our sample of rural transgender & non-binary people. This may be because provider choice was important to this group, and some insurance plans limit provider choice. In fact, of the 15% of our sample that reported changing their insurance or gaining new insurance under the Affordable Care Act, one in five said that their new insurance allowed them to choose a provider that was more LGBT-friendly. As the insurance structure of this country continues to change, we may see changes in the interaction between insurance status and health care utilization.

It is important to note several limitations of the current study. First, although rural areas nationwide are 78% white (compared to 64% of the U.S. overall [65]), this sample still lacked the expected racial and ethnic diversity in the target rural population. This may be due to a combination of factors including demographics of internet users in rural areas, method of recruitment via Facebook, or specific characteristics about the banner ads used. In addition, the social environment in rural areas may vary significantly by state or region of the country, but our study was not powered to detect these variations. Also because of the method of recruitment, our sample skewed towards respondents who are more out and who displayed their orientation or LGBT-related interests on a major online social network.

In addition, the current survey collected data on how respondents identified their own sexual orientation and gender identity, not on their sexual behavior. Thus, the sample excluded individuals who may engage in same-sex sexual behavior but do not identify as part of the LGBT community. In addition, this survey did not collect data on gender expression or how others perceived respondents’ gender. It is likely that individuals who do not conform to traditional gender roles in their everyday appearance experience more stigma, and rates of disclosure vary for these individuals as well [34]. Relatedly, due to sample size, an extraordinary amount of diversity with regards to transgender & non-binary gender identities and lived experiences was combined into one group for analysis. Despite the diversity within this group, important differences emerged between the transgender & non-binary group compared to the groups of cisgender men and women that demand further study.

Further, data analysis deviated from common practice by dividing stigma scores into domains (below approximately the 25^th^ percentile, approximately 25^th^-75^th^ percentile, and approximately 75^th^ percentile and above). Although this method was devised as a manner for determining whether a dose-response relationship was present between stigma scores and health outcomes, the determination of correlations between those variables that lie outside of the present division was restricted.

Lastly, the desired outcome variable was a singular measure of utilization of primary health care. In order to create this variable in an objective and reproducible way, the health score weighted all tests and vaccinations equally despite differences in ease of obtaining each test (including, but not limited to, factors such as time commitment, provider availability, and cost).

The study findings overall paint a picture of how stigma and outness influence rural LGBT people’s utilization of health care, beyond simply insurance status and geographic constraints. Our findings that stigma and outness are both related to utilization of care support the trend of increasing LGBT-health competency among current providers and incorporating these aspects of health care into medical education. By increasing knowledge and by promoting LGBT-inclusion within health care spaces, especially related to transgender health, stigma can be overcome and barriers to optimal primary care utilization can be dismantled. Other areas identified for further study include provider education on creating a safe environment for LGBT patients, patient education on the benefits of disclosure of sexual orientation and/or gender identity, and the relationship between risk factors, chronic disease, and poor prevention behaviors in LGBT populations.
